# Making and breaking symmetries in mind and life

**DOI:** 10.1098/rsfs.2023.0015

**Published:** 2023-04-14

**Authors:** Adam Safron, Dalton A. R. Sakthivadivel, Zahra Sheikhbahaee, Magnus Bein, Adeel Razi, Michael Levin

**Affiliations:** ^1^ Center for Psychedelic and Consciousness Research, Department of Psychiatry and Behavioral Sciences, Johns Hopkins University School of Medicine, Baltimore, MD 21224, USA; ^2^ Institute for Advanced Consciousness Studies, Santa Monica, CA 90403, USA; ^3^ Cognitive Science Program, Indiana University, Bloomington, IN 47405, USA; ^4^ VERSES Research Lab, Los Angeles, CA 90016, USA; ^5^ Departments of Mathematics, Physics and Astronomy, and Biomedical Engineering, Stony Brook University, Stony Brook, NY 11794-3651, USA; ^6^ David R. Cheriton School of Computer Science, University of Waterloo, Waterloo, Ontario, Canada N2L 3G1; ^7^ Department of Biology and Department of Psychiatry, McGill University, Montreal, Quebec, Canada H3A 0G4; ^8^ Turner Institute for Brain and Mental Health, Monash University, Clayton, VIC 3800, Australia; ^9^ Monash Biomedical Imaging, Monash University, Clayton, VIC 3168, Australia; ^10^ Wellcome Centre for Human Neuroimaging, University College London, London WC1N 3AR, UK; ^11^ CIFAR Azrieli Global Scholars Program, CIFAR, Toronto, Ontario, Canada M5G 1M1; ^12^ Tufts Center for Regenerative and Developmental Biology, Tufts University, Medford, MA 02155-5801, USA; ^13^ Allen Discovery Center, Tufts University, Medford, MA 02155-5801, USA

**Keywords:** symmetry, complex systems, biological physics, machine learning‌

## Abstract

Symmetry is a motif featuring in almost all areas of science. Symmetries appear throughout the natural world, making them particularly important in our quest to understand the structure of the world around us. Symmetries and invariances are often first principles pointing to some lawful description of an observation, with explanations being understood as both ‘satisfying’ and potentially useful in their regularity. The sense of aesthetic beauty accompanying such explanations is reminiscent of our understanding of intelligence in terms of the ability to efficiently predict (or compress) data; indeed, identifying and building on symmetry can offer a particularly elegant description of a physical situation. The study of symmetries is so fundamental to mathematics and physics that one might ask where else it proves useful. This theme issue poses the question: what does the study of symmetry, and symmetry breaking, have to offer for the study of life and the mind?

## 1. Introduction

When old age shall this generation waste,

Thou shalt remain, in midst of other woe

Than ours, a friend to man, to whom thou say'st,

‘Beauty is truth, truth beauty,—that is all

Ye know on earth, and all ye need to know’.

—from Ode on a Grecian Urn, by John Keats

Symmetry is a motif featuring in almost all areas of science. Symmetries appear throughout the natural world, making them particularly important in our quest to understand the structure of the world around us [1,2]. Symmetries and invariances are often first principles pointing to some lawful description of an observation, with explanations being understood as both ‘satisfying’ and potentially useful in their regularity [3–5]. The sense of aesthetic beauty accompanying such explanations is reminiscent of our understanding of intelligence in terms of the ability to efficiently predict (or compress) data [6,7]; indeed, identifying and building on symmetry can offer a particularly elegant description of a physical situation. The study of symmetries is so fundamental to mathematics and physics that one might ask where else it proves useful. This theme issue poses the question: what does the study of symmetry, and symmetry breaking, have to offer for the study of life and the mind?

In this diverse collection of articles, we explored the roles of symmetry in complex adaptive systems across a multitude of scales—from the emergent dynamics of biophysical systems and their underlying mechanisms, to the shaping of adaptations through ontogenic and phylogenetic processes. Across these theoretical and empirical explorations, we hoped to demonstrate that the study of symmetries may illuminate fundamental properties of living and intelligent systems. Towards this end, we considered a broad range of perspectives on (a)symmetries, exploring the extent to which intersections may be found between seemingly disparate phenomena.

One of the most powerful applications of symmetry-related concepts is found in *gauge theory* [8]. Whenever a physical theory has a redundant quantity, meaning a quantity that leaves a system's dynamics invariant with respect to local changes in the value of that quantity (a ‘frame of reference’ or ‘gauge’), we can understand that quantity as a kind of abstract symmetry recorded in what is called a gauge field [9–11]. Deformations of gauge fields are understood as ‘fictitious’ forces; these forces restore the local symmetry of quantities that are dynamically invariant, by recording the system's interactions with the field of possible gauges for that quantity. Gauge theories provide a general way of modelling physical systems. Notable use-cases include general relativity's handling of gravity as the curvature of space–time and models of the attractive and repulsive forces in electromagnetic fields. These theories are so far-reaching that the word ‘fictitious’ may potentially be left out of descriptions of these emergent forces, as it may be the case that there are no other kinds [12].

Gauge-theoretic perspectives on biophysics have been suggested in the past, especially in the context of brains as goal-seeking systems, guided by hierarchical information processing and prediction-error minimization. This is one aspect of the view presented by the free energy principle and active inference (FEP-AI) [9,13–15]: the attracting states of nervous systems are understood as entailing predictions, where the consistent realization of these predictions can be viewed as the preservation of goal states, contingent on particular symmetries being enforced by a gauge field governing those dynamics. The importance of symmetry reaches deep into the functional aspects of the brain: mental causation may be understood as a kind of ‘fictitious’ force over neural dynamics (especially with respect to perception and action) [16], and a ‘symmetry theory of valence’ has even been proposed, in which pleasure and pain may best be understood as the degree to which mental systems transition to more or less symmetric states in some appropriate sense [17–19]. In this issue, questions such as ‘along which dimensions are symmetries most important for nervous system functioning (e.g. connectomic resting-state networks as reflecting harmonic functions) [20]?’ and ‘could such organizational principles be evidenced by responses to different forms of music [21], or fractal-structured visual stimuli [22], or potentially the phenomenology associated with psychedelic states [23,24]?’ were asked and potentially answered.

Intriguingly, symmetries may play yet another distinct role in mental causation with respect to the phenomenon of symmetry breaking, where irreversible processes and arrows of time may be required for establishing the conditions for realizing cognitive work cycles [25,26] involving varying degrees of energy expenditure and various forms of either mental ‘effort’ or ‘flow’ [27–29]. These particular temporal (a)symmetries play out in the context of the principle of detailed balance [27,30] and the breaking of detailed balance. Given that many interesting biological systems do not satisfy the detailed balance property, and that breaking detailed balance (in particular, the presence of non-zero circulation) can be described using gauge theory [31], this is an interesting direction to consider in our search for symmetries [32].

The role of symmetries as perceptual invariants and inductive biases has also been identified in machine learning [33,34]. Could these physics-inspired algorithms shed light on the computational principles underlying the remarkable intelligence of biological systems?

Could the functions of symmetries in human perception and learning help inspire advances in artificial intelligence? To what extent can the study of conservation laws of nature provide more powerful and interpretable approaches to machine learning [35,36]? Indeed, when we talk about the preconditions for ‘System 2’ cognition with the power of conceptual understanding through abstraction [37,38], or even our ability to generate stable percepts, are such phenomena best understood as kinds of informational symmetries? In a different direction, we may ask: to what extent does the preference for symmetry mentioned earlier come ‘for free’ via predictive coding, where symmetric structures may be easier to predict or compress, and where efficient prediction-error minimization or compression constitutes a foundation for valence for living organisms [39–41]?

We believe these same principles apply to morphogenesis and self-organization, based on their relations to predictive coding [42], potentially offering a way to understand the kinds of unusual causation observed in living organisms as complex adaptive systems. Perhaps we may even think of pre-theoretic intuitions relating to the nature of living phenomena, where the notion of ‘elan vital’ and ‘life force’ may receive some (limited) support from abstract formalisms [43,44]. Notably, the reliable creation (and regeneration) of particular forms over the course of development has been described in terms of ‘morphogenetic fields' [45]. Some models suggest the ability of biophysical systems to construct and preserve their phenotypes can be understood as being governed by a kind of force field over an information geometry, generating particular phenotypic forms as attracting states [13]. The idea that gauge-theoretic forces could be understood as governing not just ontogeny, but also phylogeny as a free energy minimizing process, is an attractive one—especially where development is itself understood as a peculiar kind of evolution [46–50].

This collection was also inspired by the various roles symmetry can play in determining the functional properties of biological systems. Motivating questions in this direction are plentiful. How is it that symmetry breaking occurs with respect to laterality in biological systems [51–54]? How is molecular chirality, or asymmetry with respect to *direction* (present in almost all cells) amplified into body-wide asymmetry with respect to organ *position relative to the midline* in metazoan development? What is the functional significance of asymmetries in the organization of nervous systems [55–65], and do they sometimes reflect a *lack* of organismal fitness [66,67]?

The goal in asking such a broad range of questions was to look for invariances across the different ways in which the concept of symmetry can be used to characterize the natural world. While only some of these questions were directly addressed by contributors to this theme issue, the contributions we received set the stage to think much more deeply about the origins of life, consciousness and even the nature(s) of time. We are honoured to have received these contributions. Below we will attempt to provide some high-level summaries of the articles in this collection, largely drawing upon the authors' descriptions of their own work, with some of our own speculations and interpretation interspersed. Considering the profound nature(s) of these issues, there is no way we can do these authors justice with our editorial, and so we refer interested readers to the original papers.

## 2. Summary of contributions

With ‘Symmetry-simplicity, broken symmetry-complexity’, David Krakauer [68] provides a beautiful introduction to many of the core themes of this collection. He describes how complex phenomena are made possible when physical symmetries are broken and selected ground states perform mechanical work and store adaptive information. Meditating on the 50th anniversary of the groundbreaking article by Philip Anderson, ‘More is different’ [69], Krakauer describes how emergence, frustrated random functions, autonomy, and generalized rigidity characterize the nature(s) of complexity.

With these foundations in mind, ‘A third transition in science?’ by Stuart Kauffman and Andrea Roli [70] argues against the standard ‘Newtonian paradigm’ in which relevant variables and governing laws (or master equations) of systems may be clearly identified, with boundary conditions defining phase spaces over which all possible values (and their actions) are determined and fixed *a priori*, outside of time. These authors argue that such an approach is inadequate for the inherently time-dependent evolution of organisms in complex environments like our biosphere, wherein living cells exhibit ever-new adaptations, achieve constraint closure and construct themselves via evolutionary selection—leading to genuinely new possibilities that emerge from the edge of the adjacent possible. Their ultimate conclusion is that a ‘true’ phase space is necessarily undefinable using any mathematical or formal analytic tools, dashing hopes for theories of everything and the ‘Pythagorean dream’ which would attempt to capture the essence of all phenomena in terms of quantitative and symbolic terms. Based on these considerations, they suggest that this new major transition in the evolution of science may allow us to understand the nature(s) of emergence and the creativity of an evolving biosphere. In the context of this collection, by identifying natural systems as ‘Kantian wholes’, we may think of the evolution of *parts* to support the functions of *wholes* as self-organization in the sense described above: that of gauge forces and morphogenic fields.

In contrast with this anti-reductionist manifesto, with ‘Bayesian mechanics: a physics of and by beliefs', Maxwell Ramstead *et al*. [71] introduce a program attempting to do what many would consider be impossible: creating a general systems theory capable of describing all ‘things’ (in the sense of objects individuated from an environment) within a single formal modelling framework. This landmark paper ushers in a recasting of the FEP in terms of gauge theory, providing bridges between fundamental physics and dynamical systems perspectives on the FEP. Beginning from a model of a system in terms of a particular partition, wherein trajectories of the internal states of a system encode the parameters of beliefs about external states/processes, the core tenets of the FEP are rederived from the principle of maximum entropy, revealing a duality between the two. The authors discuss how, in this framework, mechanical theories can be specified for systems that ‘look as if’ they are estimating posterior probability distributions over the causes of their sensory states. The ‘inferential dynamics' [72,73] of interacting systems are set up in different classes of model described as path-tracking, mode-tracking and mode-matching. The authors then formulate a gauge-theoretic description of those inferential dynamics by using the description from maximum entropy. While this new viewpoint on complex and interacting systems will likely inspire debate, the creation of such a formal modelling framework has implications which are difficult to overstate. Most relevant to this issue is its interdisciplinarity: offering a unifying theme to be found throughout these heterogeneous domains of knowledge presents the opportunity to generate new insights, and the means by which they may be realized [74,75].

The potential scope (and impact) of such all-encompassing theories is directly supported by ‘Free energy and inference in living systems’ by Chang Sub Kim [76], which describes organisms as non-equilibrium steady state systems, self-organizing via spontaneous symmetry breaking and undergoing metabolic cycles with broken detailed balance in the environment. He goes on to suggest bridges between the principle of homeostasis as the regulation of biochemical work constrained by physical free energy costs—cf. flux balance analysis [77]—and allostasis as Bayesian inference facilitated by informational free energy, with perception and action understood in terms of the FEP. Here, brains act as a ‘Schrödinger's machine’ that minimizes sensory uncertainty. Further, in line with the aforementioned ‘Bayesian mechanics’, the author describes how optimal trajectories in neural manifolds may induce dynamic bifurcations between neural attractors in the process of active inference.

The potential explanatory (and clinical) utility of dynamical perspectives on minds in terms of bifurcating attractors is clearly expressed in ‘The lack of temporal brain dynamics asymmetry as a signature of impaired consciousness states' by Elvira García Guzmán *et al*. [78] Beginning with an intriguing discussion, the ways in which complex adaptive systems must find ways to maintain themselves far from thermodynamic equilibrium, the authors go on to present a framework based on temporal asymmetry as a measure of non-equilibrium dynamics. The authors further detail how machine learning techniques can be used to establish an ‘arrow of time’ based on the reversibility of empirically measured time-series. Fascinatingly, decreases in asymmetry and non-stationarity of brain signals were found to be characteristics of impaired consciousness states, demonstrating how highly abstract conceptual frameworks can also end up being highly practical. In this case, a deep understanding of the nature(s) of (a)symmetries in mind may yield powerful tools for studying consciousness in both fundamental and translational research.

A further beautiful example of the power of dynamical systems perspectives on brain and mind can be found in ‘Neuromodulatory control of complex adaptive dynamics in the brain’ by James Mac Shine [79], who asks a key question: how can the massive complexity of nervous systems be brought under sufficiently tight control to coordinate adaptive behaviour? Shine suggests neurons are balanced close to critical point phase transitions, where small perturbations to neuronal excitability lead to nonlinear changes in overall neural activity, in order to realize this capacity to be coordinated. He describes how various portions of the brain's ascending arousal system provide a diverse set of heterogeneous control parameters that can be used to modulate the excitability and receptivity of target neurons. These mechanisms provide control variables and critical order parameters with respect to the topological complexity of neural networks and their dynamics, which govern complex adaptive behaviours. Once again, in addition to its theoretical import, this work likely has practical consequences—describing ways in which nervous systems may (or may not) be able to operate cohesively, as integrated systems capable of adaptatively responding to a wide range of events. Criticality, and self-organization to near-critical regimes, is important for more than just flexible brain functioning [80], but potentially represents a ‘universality class’ in that it may be a hallmark of all complex adaptive systems capable of persisting in a complex and changing world. Thus, such principles of (near-)critical control may apply to more than just brains, but to all cybernetic systems, ranging from the intelligent functioning of multicellular organisms as wholes to the functioning of individual cells.

Along these lines, ‘The scaling of goals via homeostasis: an evolutionary simulation, experiment and analysis' by Léo Pio-Lopez *et al*. [81] demonstrates how fluctuating stress levels—cf. stochastic resonance and low rattling [82,83]—may allow for surprising levels of intelligence from coordinating sub-agents (here, in the context of a morphogenic process). The authors ask the question: what evolutionary dynamics enable individual cells to integrate their activities, resulting in the emergence of a novel, higher level intelligence with goals and competencies that belong to *it* and not to its parts? They describe a system consistent with the ‘TAME’ framework, in which evolution harnesses the collective intelligence of cells during morphogenesis of the body to develop traditional behavioural intelligence, scaling up goal states at the centre of homeostatic processes [84]. Using the classic French flag problem as a model of the establishment and maintenance of a particular symmetry (a body-wide positional axis), the authors confirmed predictions that emergent morphogenetic agents could use a combination of local and global signalling to achieve target morphologies, recover from perturbation, achieve long-term stability and even suddenly remodel long after the system stabilizes (a hallmark of near-critical systems). They further tested these predictions on a genuine biological system, observing similar phenomena in regenerating *Planaria* (flatworms), suggesting that these principles of intelligence arising from multi-scale competent sub-agents may provide a powerful explanatory framework for understanding the principles of intelligence underlying all living systems, and potentially our attempts to engineer systems that operate according to similar principles.

In ‘Embodied cognitive morphogenesis as a route to intelligent systems’, Bradly Alicea *et al.* [85] provide a powerfully compelling account of the ways in which morphogenetic symmetry breaking produces specialized organismal subsystems, and how this serves as a substrate for the emergence of autonomous behaviours with properties related to acquisition, generativity and transformation. They describe this embodied cognitive morphogenesis as providing a means of bridging an ‘embryological view’ (emphasizing coordinated gene expression, cellular physics, and migration as the basis for phenotypic complexity) with a more enactivist perspective (centring on informational feedback between organisms and their environment is key to the emergence of intelligent behaviours). They further outline their work with generic organismal agent modelling—involving tensegrity networks, differentiation trees and embodied hypernetworks—providing a means to identify the context for various symmetry-breaking events in developmental time. Theirs is a richly detailed framework that integrates a diversity of concepts, including modularity, homeostasis, 4E (embodied, enactive, embedded and extended) cognition and more.

Contemplating the asymmetric production of forms in nature, with ‘Chiral conformity emerges from the least-time free energy consumption’, Arto Annila [86] describes chiral symmetry breaking (‘handedness’, or geometric rotational asymmetry) across multiple systems, ranging from biophysical processes to the disproportional generation of matter and antimatter in the creation of the universe. The author argues that such symmetry breaking may not necessarily involve initial biases with respect to some underlying generative process. Rather, an analogy may be drawn with some aspects of handedness standards in societies, which have ‘evolved over time to make things work’. The author goes on to draw connections between work as the universal measure of transferred energy, with universal principles of free energy minimization, and its relationships with the principle of maximum entropy production (as described earlier in this collection). The author further argues for an ontology that describes everything in terms of ‘quanta of action’, providing further bridges to the FEP (as a framework of systems actively inferring themselves into being), and for a universal law in which flows of energy naturally select some structures over others, based on their capacity to consume free energy in the least time [87]. This may be thought of as another convergent line of support for the approach of Bayesian mechanics, based on its connections to the principle of maximum calibre [88]. Ultimately the author concludes that it is ‘meaningless’ to speculate about life's origins, since ‘thermodynamics makes no distinction between animate and inanimate’.

However, if approached with care, perhaps such questions may not be as meaningless as they might seem. In ‘Mixed anhydrides at the intersection between peptide and RNA autocatalytic sets: evolution of biological coding’, Stuart Kauffman and Niles Lehman [89] offer a proposal for the origins of biological coding as a semiotic relationship between chemical information stored in one location that links to chemical information stored in a separate location. In their proposal, coding originates from cooperation between two, originally separate, collectively autocatalytic sets, which (via pressures to eliminate energetic waste) eventually results in a 1 : 1 relationship between single amino acids and short RNA pieces—establishing the ‘genetic code’. The idea of Kantian wholes is introduced again, where every stage in the evolution of coding is driven by the downward selection on the components of a system, according to a kind of criterial causation [90]. On this view, the separation between *code* and *coded* was a prerequisite for robust cumulative evolution [91–93] and thus may be ‘synonymous with life as we know it’.

In ‘As without, so within: how the brain's temporo-spatial alignment to the environment shapes consciousness', Georg Northoff *et al*. [94] consider symmetries with respect to modes of synchronization between brain and environment. With the ‘temporo-spatial theory of consciousness’, temporo-spatial alignment provides a mechanism by which the brain adapts to—and coordinates its neuronal activity with—various interoceptive (bodily) and exteroceptive (environmental) stimuli. A three-layer, conceptual, neuro-phenomenal model for consciousness is proposed; this is constituted by various lengths of the brain's intrinsic neuronal timescales, each corresponding to phenomenal layers of consciousness, such as the environmental background and specific contents in the foreground of awareness. These layers are shared between the brain's neuronal activity and consciousness, providing their ‘common currency’ on dynamical grounds coupled to the environment. Through temporo-spatial alignment this suggests ways in which various forms of mental representation may emerge as a kind of resonant matching (or entrainment), which offers analogies to both thermodynamic and informational free energy gradients. These ideas are also considered in terms of the closely related integrated world modelling theory of consciousness, which attempts to bring together multiple models within the overarching framework of the FEP [95,96].

In ‘Symmetry and complexity in object-centric deep active inference models', Stefano Ferraro *et al*. [97] describe autonomous robotic systems designed according to the principles of the FEP, wherein agents achieve coherent perception—and take intelligent actions—by employing mental models of objects that exploit symmetries in shape and appearance. Such ‘object-ness’ is often described as part of inborn ‘core knowledge’, by which biological learners are capable of efficiently bootstrapping sophisticated mental functions with so little training data [98,99]. This work, however, demonstrates how such capacities may be learned without clear innate inductive biases directly related to learning object models. Rather, these agents learn and act by minimizing an upper bound on their surprisal (i.e. their free energy) with respect to a generative model describing (and governing) interactions with their environment. This free energy objective functional decomposes into accuracy and complexity terms, inducing pressure on agents to favour simpler models that can accurately explain sensory observations. As a result of this set-up, inherent symmetries of particular objects also emerge as (complexity-minimizing) symmetries in latent state spaces of generative models that minimize free energy. These object-centric representations further allow for novel object views to be predicted as the agent moves and changes its perspective on the world. The principal axes of such symmetries were observed as principal components explaining significant amounts of variance for objects within latent space, which were not just elegantly explanatory, but also functional, in that exploiting these symmetries allowed for better generalization in the context of robotic manipulation. Intriguingly, shared latent space representations have also been associated with consciousness theories based on world modelling [5,95,96]. There, perceptions are generated as the iterative prediction of likely system-world configurations, conditioned on causal world models whereby embodied agents select actions predicted to realize value (or minimize expected free energy). Perhaps more relevant to this theme issue, if the identification of symmetric forms is an essential part of autonomous functioning (and a means of reducing the complexity of internal models), then it may be no surprise that we often search for (and construct) such forms in the world.

The power of identifying symmetries for adaptive functioning is compellingly demonstrated with ‘Emergence of common concepts, symmetries, and conformity in agent groups—an information-theoretic model’, in which Marco Möller and Daniel Polani [100] argue that leveraging symmetry is the principle underlying the efficient and accurate formation of shared representations of the world. The authors demonstrate, in a simulation of agents capable of perception–action loops, that when some common structure is appreciated by all agents at once, it is usually one rooted in some symmetry of the environment. In these models of a simple environment, individuals extract representations through an information maximization principle, which differ across agents to varying degrees. Drawing upon a variant of the information bottleneck principle [101–103], they extract a ‘common conceptualization’ of the world for this group of agents, which is shown to emphasize higher regularities (or symmetries) of the environment, as compared to the individual representations. Möller and Polani further examine the identification of symmetries in the environment both with respect to ‘extrinsic’ (allocentric) operations on the environment as well as with respect to ‘intrinsic’ operations corresponding to reconfigurations from the point-of-view of the agent's particular embodiment-embedding. The authors note how the intrinsic perspective supports more efficient coordination of concepts, which further support generalization and transfer across learning environments. In the light of the effectiveness of group equivariant neural networks, which are known to exploit representational symmetries in data [104,105], this is a particularly relevant contribution to our issue. It is tempting to think of its potential extensions to modelling groups as collective minds [106,107], as well as minds as collections of sub-agents [108–110]. One may even wonder whether identifying symmetries, as points of coordination among competing and cooperating processes, may provide one of the major functional or adaptive roles of consciousness—especially in the context of the information bottleneck or cybernetic control [111–113].

Finally, we come full circle, examining the question of how there may be space for our meanings in the physical world [114]. In ‘Reflections on the asymmetry of causation’, Jenann Ismael [115] helps to guide us through a vision in which scientific understanding and the ‘manifest image’ may be not just reconcilable [16,116], but mutually enriching. She begins by reminding us how causation is a paradigmatic example of an objective worldly relation, even *the* fundamental ordering relation of the world. She then considers Bertrand Russell's suggestions that the concept of causation ought to be replaced with time-symmetric laws of temporal evolution and goes on to explore the question of how time asymmetries emerge from otherwise symmetric underlying dynamics. While there may be no place for notions of time or cause in fundamental physics, the asymmetry of causation is arguably the most important kind of symmetry breaking for our experience of the natural world. Ismael asks the question ‘[W]hat precisely, is the status of the causal arrow, assuming a thermodynamic gradient and the interventionist account of causation?’ She then provides a compelling answer to this question, by showing how an objective asymmetry can be rooted in interventionist causal pathways that propagate influence into the future, but not into the past. She supports this argument by suggesting that the present macrostate of the world screens off probabilistic correlations to the past, in the presence of a low entropy boundary condition and under a particular agent-centred coarse-graining of the world. However, this agent-dependent carving up of the world does not leave us with arbitrary perspectivism, but instead demands a certain shared sensemaking. It is neither the singular vision of the Newtonian paradigm, nor the view-from-nowhere of Laplace's demon, nor a capricious relativism. Rather, it is an ecumenical, human place, where we may find space for things such as beauty and meanings, and—perhaps—even truth.

## 3. Conclusion

Each of these articles presents an interesting take on a symmetry or asymmetry relevant to complex adaptive systems. We believe that the study of such systems, which includes many of the most interesting questions in twenty-first-century physics—ranging from soft matter and active matter, to living and intelligent systems, to neural dynamics and consciousness—is unexpectedly rich in symmetries. While not all these articles are conceptualized in terms of the FEP, we nonetheless believe that it provides a golden thread stretching through this collection, complementary and deeply entwined with the application of principles of symmetry to the world of complex systems. However, no matter the particular formalism within which we ask and answer these questions, one thing is clear: the search for symmetry is a productive one that will likely prove a guiding principle in these areas—just as it has been throughout history in other areas of mathematics and physics.

## Data Availability

This article has no additional data.
